# Crosssectional Assessment of Bone Mass Density in Adults with Hepatitis B Virus and Hepatitis C Virus Infection

**DOI:** 10.1038/s41598-019-41674-4

**Published:** 2019-03-25

**Authors:** Yuan-Yuei Chen, Wen-Hui Fang, Chung-Ching Wang, Tung-Wei Kao, Yaw-Wen Chang, Hui-Fang Yang, Chen-Jung Wu, Yu-Shan Sun, Wei-Liang Chen

**Affiliations:** 1 Department of Internal Medicine, Tri-Service General Hospital Songshan Branch; and School of Medicine, National Defense Medical Center, Taipei, Taiwan Republic of China; 2 Division of Family Medicine, Department of Family and Community Medicine, Tri-Service General Hospital; and School of Medicine, National Defense Medical Center, Taipei, Taiwan Republic of China; 3 Division of Geriatric Medicine, Department of Family and Community Medicine, Tri-Service General Hospital; and School of Medicine, National Defense Medical Center, Taipei, Taiwan Republic of China; 4 Graduate Institute of Clinical Medical, College of Medicine, National Taiwan University, Taipei, Taiwan Republic of China; 5 Division of Family Medicine, Department of Community Medicine, Taoyuan Armed Forces General Hospital, Taoyuan, Taiwan Republic of China

## Abstract

Osteoporosis is one of the major complications in chronic hepatitis B virus (HBV) and hepatitis C (HCV) infection. However, few studies had examined the relationship between hepatic viral infection with bone loss. Our aim was to investigate the association between hepatic viral infection with bone mineral density (BMD) in a cross-sectional study. Participants who attended the health examinations at the Tri-Service General Hospital (TSGH), Taiwan, were enrolled in the study. Diagnosis of viral hepatitis was confirmed by the serum viral markers of hepatitis B surface antigen (HBsAg) and anti-HCV, and BMD measurement was performed by the bone densitometry. Subjects were divided into four groups by the presence of viral markers. The association between hepatic viral infection and BMD was examined by a multivariate linear regression model. HBV infection was inversely associated with BMD after full adjusting with *β* values of −0.17 (95% CI: −0.29, −0.05) (p < 0.05). The relationship remained significant in males (*β* = −0.16, 95% CI = −0.31, −0.01) (p < 0.05). In subjects with body mass index less than 30 HBV infection was associated with reduced BMD (β = −0.16, 95% CI = −0.29, −0.02) (p < 0.05). However, HCV infection was only associated with an increase in BMD in patients with BMI less than 30 (β = 0.17, 95% CI = 0.21, 0.32) (p < 0.05). Chronic HBV infection was significantly associated with reduced BMD in males. The impact of viral hepatitis on bone health deserves further investigation for the potential pathophysiological mechanisms.

## Introduction

Osteoporosis, which diagnosed by measurement of bone mineral density (BMD), is a common health problem worldwide due to its high risk for fractures, morbidity and mortality^1^. The etiology of osteoporotic fracture is complex and multifactorial, including low bone mass, ethnicity, hormonal factors, drug, and low vitamin D deficiency^2^. Chronic liver disease is suggested to be a major risk factor of bone loss and osteoporosis due to decreased bone formation or increased bone resorption^3,4^. Previous studies had reported osteoporosis in patients with cirrhosis, particularly caused by viral hepatitis^5,6^.

Viral hepatitis is a global health problem and as many as 365 million people had been infected with hepatitis B virus (HBV) and about 170 million people infected with hepatitis C virus (HCV) in the world^7,8^. Hepatic viral infection is one of the major etiologies of liver diseases in Taiwan and a 17.3% seroprevalence of HBV and a 4.4% seroprevalence of HCV were estimated^9^. Apart from liver diseases, both HBV and HCV infection have many extrahepatic manifestations such as hematologic, autoimmune and dermatologic disorders^10^. However, few studies had reported osteoporosis and bone loss in non-cirrhotic patients with hepatic viral infection.

The aim of the current study was to determine the association between hepatic viral infection and bone health. We investigated BMD in non-cirrhotic adults with hepatic viral infection in comparison with those without viral hepatitis in a cross-sectional analysis.

## Result

### Demographic characteristics of study population

The baseline information collected from study population was shown in Table 1. The mean age of each viral hepatitis group was 39.88 ± 13.80 (normal), 44.33 ± 13.77 (HCV), 43.44 ± 11.63 (HBV), and 45.82 ± 11.42 (HCV/HBV). Subjects with co-infection viral hepatitis had lower BMD than other groups. Those with HBV infection had an elevation in liver enzymes that aspartate aminotransferase (AST) values were higher than other groups. Characteristics including age, BMD, body mass index (BMI), creatinine (Cr), AST, albumin, high-sensitive C-reactive protein (hsCRP), thyroid stimulating hormone (TSH), history of proteinuria and alcoholic consumption had significant difference across these viral hepatitis groups.

**Table 1:** Characteristics of study sample.

Variables	Normal	HCV	HBV	HCV + HBV	P Value
**Continuous Variables, mean (SD)**					
Age (years)	39.88 (13.80)	44.33 (13.77)	43.44 (11.63)	45.82 (11.42)	<0.001
T-score	0.48 (1.42)	0.46 (1.43)	0.35 (1.41)	0.25 (1.29)	0.032
Body mass index (kg/m^2^)	23.87 (4.05)	24.01 (3.96)	24.21 (4.02)	23.72 (4.00)	<0.001
Total cholesterol (mg/dL)	185.16 (35.67)	185.09 (36.57)	185.29 (33.42)	183.41 (35.82)	0.788
Uric acid (mg/dL)	5.55 (1.48)	5.58 (1.47)	5.60 (1.44)	5.52 (1.49)	0.252
Creatinine (mg/dL)	0.81 (0.30)	0.83 (0.48)	0.83 (0.34)	0.81 (0.26)	<0.001
AST (U/L)	20.12 (11.94)	20.97 (10.87)	24.84 (29.14)	22.08 (11.87)	<0.001
Albumin (g/dL)	4.51 (0.29)	4.46 (0.27)	4.51 (0.30)	4.44 (0.28)	<0.001
HsCRP (mg/dL)	0.24 (0.51)	0.26 (0.49)	0.20 (0.48)	0.19 (0.23)	0.045
TSH (IU/mL)	2.25 (1.67)	2.28 (1.74)	2.16 (1.40)	2.53 (2.73)	0.007
**Category Variables, (%)**					
Gender (male)	21633 (51.7)	1024 (48.4)	2708 (57.9)	220 (48.4)	<0.001
Obesity (BMI > 30 kg/m^2^)	7738 (19.2)	401 (20.0)	901 (20.2)	80 (20.6)	0.076
Proteinuria	12415 (32.2)	564 (30.8)	1364 (29.9)	149 (33.2)	0.006
Smoking	4509 (28.4)	333 (30.4)	595 (28.8)	64 (28.2)	0.542
Drinking	6668 (48.0)	503 (46.4)	788 (46.0)	97 (43.7)	0.033

### Association between viral hepatitis and BMD

In Table 2, the associations between different viral hepatitis with BMD were analyzed by a multivariable linear regression model. HBV infection was inversely associated with BMD after adjusting for pertinent clinical variables with *β* values of −0.19, −0.17, and −0.17 (95% CI: −0.31, −0.06; −0.29, −0.05; −0.29, −0.05) (p < 0.05) in Model 1 to Model 3, respectively.

**Table 2:** Association between hepatic viral infection and BMD.

Variables	Model^a^ 1*β*^b^ (95% CI)	*P* Value	^*c*^ *R* ^2^	Model^a^ 2 *β*^b^ (95% CI)	*P* Value	^*c*^ *R* ^2^	Model^a^ 3 *β*^b^ (95% CI)	*P* Value	^*c*^ *R* ^2^
***BMD***									
***Healthy group***	***reference***	—	0.163	***reference***	—	0.175	***reference***	—	0.176
HCV	0.08 (−0.06, 0.22)	0.256		0.09 (−0.05, 0.23)	0.191		0.09 (−0.04, 0.23)	0.182	
HBV	−0.19 (−0.31, −0.06)	0.003		−0.17 (−0.29, −0.05)	0.006		−0.17 (−0.29, −0.05)	0.007	
HCV + HBV	−0.21 (−0.51, 0.10)	0.187		−0.19 (−0.49, 0.12)	0.225		−0.19 (−0.49, 0.12)	0.228	

^a^Adjusted covariates:

Model 1 = age + gender + BMI.

Model 2 = Model 1 + proteinuria, serum total cholesterol, uric acid, creatinine, AST, albumin, hsCRP, TSH.

Model 3 = Model 2 + history of smoking, drinking.

^***c***^***R***^2^: Adjusted R squared.

After categorizing subjects into gender difference (Table 3), the relationship remained significant in males but not females. Besides, those with HCV/HBV co-infection (*β* = −0.45, 95% CI = −0.86, −0.04) (p < 0.05) were more closely associated with reduced BMD than HBV infection (*β* = −0.16, 95% CI = −0.31, −0.01) (p < 0.05).

**Table 3:** Association between hepatic viral infection and BMD in gender difference.

Gender	Variables	Model^a^ 1 *β*^b^ (95% CI)	*P* Value	^*c*^ *R* ^2^	Model^a^ 2 *β*^b^ (95% CI)	*P* Value	^*c*^ *R* ^2^	Model^a^ 3 *β*^b^ (95% CI)	*P* Value	^*c*^ *R* ^2^
***BMD***										
Male	HCV	0.16 (−0.02, 0.33)	0.090	0.074	0.15 (−0.03, 0.33)	0.107	0.089	0.15 (−0.03, 0.32)	0.106	0.090
	HBV	−0.17 (−0.33, −0.01)	0.035		−0.16 (−0.32, −0.01)	0.047		−0.16 (−0.31, −0.01)	0.050	
	HCV + HBV	−0.48 (−0.89, −0.06)	0.025		−0.46 (−0.87, −0.05)	0.028		−0.45 (−0.86, −0.04)	0.031	
Female	HCV	−0.07 (−0.27, 0.12)	0.453	0.297	−0.07 (−0.26, 0.13)	0.510	0.297	−0.07 (−0.26, 0.13)	0.496	0.296
	HBV	−0.14 (−0.34, 0.01)	0.069		−0.16 (−0.34, 0.02)	0.083		−0.16 (−0.34, 0.02)	0.080	
	HCV + HBV	0.17 (−0.24, 0.58)	0.411		0.17 (−0.24, 0.58)	0.425		0.17 (−0.25, 0.58)	0.430	

^a^Adjusted covariates:

Model 1 = age + BMI.

Model 2 = Model 1 + proteinuria, serum total cholesterol, uric acid, creatinine, AST, albumin, hsCRP, TSH.

Model 3 = Model 2 + history of smoking, drinking.

^***c***^***R***^2^: Adjusted R squared.

In Table 4, we analyzed the associations between different viral hepatitis with BMD with or without the presence of obesity (BMI > 30 kg/m^2^). Non-obesity subjects with HBV infection remained significant among this inverse relationship (*β* = −0.16, 95% CI = −0.29, −0.02) (p < 0.05). In contrast, non-obesity subjects with HCV infection had association with increased BMD in fully-adjusted model with *β* values of 0.17 (95% CI = 0.02, 0.32) (p < 0.05). However, no significant finding was noted in obese population.

**Table 4:** Association between hepatitis viral infections and BMD with or without the presence of obesity.

Obesity	Variables	Model^a^ 1 *β*^b^ (95% CI)	*P* Value	^*c*^ *R* ^2^	Model^a^ 2 *β*^b^ (95% CI)	*P* Value	^*c*^ *R* ^2^	Model^a^ 3 *β*^b^ (95% CI)	*P* Value	^*c*^ *R* ^2^
***BMD***										
Obesity (BMI > 30)	HCV	−0.21 (−0.52, 0.10)	0.189	0.082	−0.21 (−0.52, 0.10)	0.183	0.083	−0.22 (−0.53, 0.10)	0.174	0.084
	HBV	−0.22 (−0.49, 0.05)	0.110		−0.22 (−0.49, 0.06)	0.118		−0.20 (−0.47, 0.07)	0.142	
	HCV + HBV	−0.18 (−0.94, 0.57)	0.632		−0.20 (−0.95, 0.55)	0.602		−0.16 (−0.91, 0.60)	0.687	
Non-obesity (BMI < 30)	HCV	0.15 (−0.01, 0.30)	0.052	0.160	0.17 (0.02, 0.31)	0.031	0.175	0.17 (0.02, 0.32)	0.028	0.175
	HBV	−0.16 (−0.30, −0.02)	0.021		−0.16 (−0.29, −0.02)	0.025		−0.16 (−0.29, −0.02)	0.027	
	HCV + HBV	−0.21 (−0.54, 0.12)	0.205		−0.21 (−0.54, 0.12)	0.215		−0.21 (−0.54, 0.12)	0.216	

^a^Adjusted covariates:

Model 1 = age + gender + BMI.

Model 2 = Model 1 + proteinuria, serum total cholesterol, uric acid, creatinine, AST, albumin, hsCRP, TSH.

Model 3 = Model 2 + history of smoking, drinking.

^***c***^***R***^2^: Adjusted R squared.

## Discussion

The important role of viral hepatitis in the bone health was highlighted in the current study. Subjects with HBV infection was significantly associated with reduced BMD, especially in male population. Non-obesity participants with HBV infection remained the inverse relationship, but those with HCV infection was correlated with increased BMD. To date, our study was the first to examine the associations between different viral hepatitis with BMD in a large population-based analysis composed of Taiwanese adults.

Accumulated evidence had reported the relationship between hepatic viral infection with BMD and osteoporosis. Huang *et al*. demonstrated that chronic HBV infection were associated with low BMD and increased risk of developing subsequent osteoporosis in a case-control study composed of 148 patients^11^. Bone mineral metabolism was affected by both HBV and HCV infection that high prevalence of low BMD was observed in 60 participants^12^. Lower BMD at the femur was noted among 60 untreated chronic HCV-infected patients than 59 healthy men^13,14^. Compared with these studies, our finding was based on a large population-based design which involved in 51144 subjects. In addition, we analyzed different viral hepatitis groups including normal, HCV, HBV and HCV/HBV co-infection simultaneously. Chen *et al*. had published a nationwide population-based study from the Longitudinal Health Insurance Database that HBV infection increased the risk of osteoporosis^15^. However, the diagnosis of viral hepatitis and osteoporosis was obtained from ICD-9 codes that might be influenced by wrong diagnoses or codes. The accuracy in diagnosis of osteoporosis could not validate through radiography and the same condition was existed in the diagnosis of HBV infection. In our study, BMD was detected by DEXA at the health examinations. Viral hepatitis was diagnosed by serum viral markers of HBsAg and anti-HCV at the same places.

The mechanisms for interaction between hepatic viral infection with BMD were still unclear. Inflammatory cytokines such as interleukin-6 (IL-6) and tumor necrosis factor-alpha (TNFα) dominated the pathophysiologic response to the chronic HBV infection with an increased cytokine release^16^. These cytokines could activate nuclear factor kappa-B ligand (NFkappaB) to stimulate osteoclastogenesis, which caused decreasing bone formation and increasing bone resorption increasing and led to reduced BMD^17^. The effect of vitamin D was well known on not only bone metabolism but also modulation of immune responses^18,19^. Vitamin D deficiency was involved in the pathogenesis of virus-infected liver diseases caused by HBV and HCV^20^. Previous studies had reported that insufficient vitamin D levels most likely failed to suppress HBV replication and contribute to poor clinical courses. Vitamin D deficiency contributed to altered bone mineralization and led to low bone mass^21,22^. Low serum 25(OH) D levels were associated with high parathyroid hormone (PTH) that led to increasing bone turnover and accelerated primarily cortical but also trabecular bone loss^23^. Another potential explanation was mediated by impaired insulin-like growth factor-1(IGF-1), which was caused by virus-infected liver decompensation and cirrhosis^24^. The IGF- regulatory system was critical for skeletal growth and maintenance^25^. Serum IGF-1 levels were supported to be positively associated with bone mass that inhibited osteoblast differentiation and proliferation^26^. Oncofetal fibronectin, an isoform of fibronectin produced by the matrix-producing stellate cells in the liver, is increased in individuals with HCV and a variety of liver diseases^27,28^. Oncofetal fibronectin can directly inhibit osteoblast function and contribute to decreased BMD^29^. However, since there was an association with increased BMD in HCV in males with body mass index less than 30 this could be due to an increase in another isoform called EDA that is also detected in HBV patients^27,30^. Further study is warranted to investigate the potential mechanisms for protective effect of HCV on bone mass.

Clinical statistics represented the view that male subjects had faster progression rate of chronic HBV and HCV infection than females^31^. Alward *et al*. demonstrated that female patients had higher prevalence of HBeAg clearance than males^32^. Seroconversion from HBsAg to anti-HBs and from HBeAg to anti-HBe developed more frequently in female subjects compared with in males^33^. Estradiol had been suggested to increase interferon (INF)-γ levels in lymphocytes and enhanced response of antigen-specific primary antibody in mononuclear cells of human peripheral blood^34,35^. Premenopausal females might have antibodies against HBeAg and HBsAg at a higher frequency than those in postmenopausal and males due to the antiproliferative and immunomodulatory properties of IFN-γ. Collectively, it was consistent with our finding that male population with viral hepatitis seemed to have higher inflammatory activity than females that contributed to significantly reduced BMD.

Obesity was generally linked to protective factor for skeletal health and increased BMD^36^. Several evidence had indicated that it was positively correlated with high bone mass because of increased levels of leptin, insulin, and estrogen, stimulating bone growth and inhibiting bone remodeling^37^. The protective effect of increased leptin levels on bone mass was reported in obese subjects as result of the interaction between leptin with the RANKL/RANK system^38^. It implied that the detrimental impact of hepatic viral infection on BMD tended to occur in non-obesity population, which was lacking the beneficial effect from obesity.

Although the study has strength of a large population-based survey, there were several potential limitations. First, we could not address the casuality between hepatic viral infection with BMD. A longitudinal analysis is more suitable than cross-sectional design of our study. Second, the bone-related lifestyle factors could not be confirmed, even though we tried to adjust for potential confounding factors. Third, BMD values were limited to T-score measurement at L1–L4, because, absolute density and Z-score are unavailable from the health examination. Next, study population was only composed of an Asian population, with limited ethnic diversity. Last, the precise mechanisms of hepatitis viral infection on BMD were unknown.

In conclusion, the present study highlighted the important role of HBV infection in the development of reduced BMD in a Taiwanese adult population. The association between viral hepatitis and bone health deserved further investigation for the potential underlying pathophysiological mechanisms. Strategies for preventing the detrimental impact of chronic liver diseases and longitudinal research for predicting risks of incident low BMD, even osteoporosis, were necessary.

## Methods

### Study population

Participants who attended the health examinations at the Tri-Service General Hospital, Taiwan, from 2010 to 2016 were included in the study. The comprehensive examinations included laboratory data, body composition and bone mineral density measurement, and detailed self-reported questionnaires. All protocols in the study were approved by the Institutional Review Board of Tri-Service General Hospital, Taiwan. The TSGH IRB waived the individual informed consent because these data were analyzed anonymously. All methods were performed in accordance with the relevant guidelines and regulations.

### Study design and screening program

Exclusion criteria of our study were chronic liver diseases (i.e. cirrhosis, hepatocellular carcinoma, autoimmune and genetic liver disease), celiac disease, inflammatory bowel disease, chronic kidney disease, cancer, lupus, multiple myeloma, rheumatoid arthritis, thyroid disorders, missing information (including baseline characteristic data, serum hepatic viral markers examination, and dual energy x-ray absorptiometry (DEXA)). 51144 residual subjects were categorized into four groups (normal, HCV, HBV, HCV/HBV) based on the presence of viral markers. The association between hepatic viral infection with BMD was performed by a multivariable linear regression model.

### Examination of hepatitis B/C infection

Serum viral markers of hepatitis B surface antigen (HBsAg) and anti-HCV were tested at the health examinations at TSGH. Radioimmunoassay kits (Abbott Laboratories, Chicago, IL, USA) were performed for detecting HBsAg. Anti-HCV was detected by using a 3^rd^ generation enzyme immunoassay (Abbott Laboratories).

### Measurement of BMD

BMD was measured by Dual energy X-ray absorptiometry (DEXA) in the health examinations at TSGH by using Prodigy Series X-Ray Tube Housing Assembly (GE Medical Systems Lunar 3030 Ohmeda Dr Madison, Wisconsin, USA). DEXA was the most frequently used technique for measuring BMD at various body sites. The density of the lumbar spine was measured rather than the total hip. We excluded these participants with past histories of vertebral fracture, vertebroplasty, or implants of polymethylmethacrylate cement.

### Assessments of covariates

The baseline information included demographic factors (age, gender), body composition (BMI, BMD), laboratory data [serum total cholesterol (T-CHO), uric acid (UA), Cr, AST, albumin, hsCRP, TSH], and personal history (proteinuria, cigarette smoking, alcoholic consumption). A self-reported questionnaire was used to collect age, gender and personal history. BMI was calculated by a formula that the weight divided by the square of the height (kg/m^2^) of a participant. Subjects were requested to fast at least 8 hours before health examinations for collecting blood samples. Biochemistry data was analyzed by different standard measurements. T-CHO was analyzed by an enzymatic colorimetric method (Roche Diagnostics, Indianapolis, IN, USA). The latex-enhanced nephelometry was used to detect hsCRP. UA was measured by the Hitachi 737 automated multichannel chemistry analyzer (Boehringer Mannheim Diagnostics, Indianapolis, IN, USA). Cr was measured by the uncompensated Jaffe method with the alkaline picrate kinetic test. Participants who had cigarette smoking were derived from asking the question “How many packs do you smoke per day?”. Alcoholic drinking was defined by a self-report questionnaire ranging from a drinking frequency of “never” to “alcohol consumption”. Proteinuria was determined by dipstick test, a useful tool for diagnosing changes in urine sample in standard urinalysis.

### Statistical analysis

We classified subjects into different hepatic viral infection and compared the distribution of characteristics and covariates across subgroups by using ANOVA for continuous variables and the chi-squared test for categoric variables. Statistical significance was defined as a two-sided *p*-value of ≤0.05. Multivariable models were adjusted as follows. Model 1: age, gender and BMI. Model 2: Model 1 + proteinuria, serum total cholesterol, uric acid, creatinine, AST, albumin, hsCRP, and TSH. Model 3: Model 2 + history of cigarette smoking and alcoholic consumption. A linear regression model was performed for the association between hepatic viral infection and BMD. Analyses in the current study were conducted by Statistical Package for the Social Sciences, version18.0 (SPSS Inc., Chicago, IL, USA) for Windows.
